# Lipoprotein lipase responds similarly to tinzaparin as to conventional heparin during hemodialysis

**DOI:** 10.1186/1471-2369-11-33

**Published:** 2010-12-06

**Authors:** Dana Mahmood, Maria Grubbström, Lennart DI Lundberg, Gunilla Olivecrona, Thomas Olivecrona, Bernd G Stegmayr

**Affiliations:** 1 Department of Internal Medicine, County Hospital in Östersund, SE-83131 Östersund, Sweden; 2 Department of Internal Medicine, University Hospital, SE-90185, Umeå, Sweden; 3 Department of Medical Biosciences, University of Umeå, SE-90187 Umeå, Sweden

## Abstract

**Background:**

Low molecular weight (LMW) heparins are used for anticoagulation during hemodialysis (HD). Studies in animals have shown that LMW-heparins release lipoprotein lipase (LPL) as efficiently as unfractionated (UF) heparin, but are less able to retard hepatic uptake of the lipase. This raises a concern that the LPL system may become exhausted by LMW-heparin in patients on HD. We have explored this in the setting of clinical HD.

**Methods:**

Twenty patients on chronic hemodialysis were switched from a primed infusion of UF-heparin to a single bolus of tinzaparin. There were long term follow up of variables for the estimation of dialysis efficacy as well as of the LPL release during dialysis and the subsequent impact on the triglycerides.

**Results:**

The LPL activity in blood was higher on tinzaparin at 40 but lower at 180 minutes during HD. These values did not change during the 6 month study period. There were significant correlations between the LPL activities in individual patients at the beginning and end of the 6 month study period and between the activities on UF-heparin and on tinzaparin, indicating that tissue LPL was not being exhausted. Triglycerides were higher during the HD-session with tinzaparin than UF-heparin. The plasma lipid/lipoprotein levels did not change during the 6 month study period, nor during a 2-year follow up after the switch from UF-heparin to tinzaparin. Urea reduction rate and Kt/V were reduced by 4 and 7% after 6 months with tinzaparin.

**Conclusion:**

Our data demonstrate that repeated HD with UF-heparin or tinzaparin does not exhaust the LPL-system.

## Background

During hemodialysis (HD) the patient must receive anticoagulation. According to the traditional protocol this is given as a primed infusion of UF-heparin. A newer approach, that has practical advantages, is to give a single bolus of LMW-heparin [1,2]. This is possible since the LMW preparations are cleared more slowly from blood; half-lives of 2 - 6 hours have been reported compared to only about 1 h for UF-heparin. Furthermore, whereas the pharmacodynamics of UF-heparins shows large differences between patients, the elimination kinetics for LMW-heparins is more predictable and therefore safe in most situations. One consideration when comparing the two preparations is that heparin releases the enzyme lipoprotein lipase (LPL) from its binding sites at the vascular endothelium into the circulating blood [3]. This leads to accelerated degradation of the enzyme which is taken up from blood into the liver, and this in turn leads to a period of relative LPL depletion during which the metabolism of triglyceride (TG)-rich lipoproteins is slowed down [4,5]. Studies in experimental animals [4] and in human subjects [6] indicate that this is more marked with LMW-heparins compared to UF-heparin. The local clinical HD unit recently decided to switch from UF-heparin to tinzaparin (one of the LMW-heparins that is commonly used for HD [7-9]). In conjunction with this, we decided to carry out a quality assurance investigation with emphasis on effects of the different heparins on the LPL system and clinical variables in the setting of chronic HD. A major question was if the LPL system may become exhausted by LMW-heparin in patients on HD.

## Methods

### Patients and study protocol

The study included twenty patients who had been on chronic HD for at least 3 months (range 3-72 months). Baseline data are given in table 1. The reason for dialysis was diabetes mellitus in six patients, vasculitis in four, interstitial nephritis in three, polycystic kidney disease in two, glomerulonephritis in two, and one each with nephrosclerosis, post renal obstructive problems, and myeloma associated amyloidosis. Eleven of the patients were on statin medication, with unchanged dose, throughout the study. Before the study all patients were on dialysis with primed infusion of UF-heparin (Leo Pharma, Ballerup, Denmark) as anticoagulant (table 2). Data were not obtained during the entire study period for four subjects; two died (one due to progressive myeloma, one due to congestive heart failure); two received renal transplants. The Regional Ethical Review Board in Umeå, Sweden, approved the study. Informed consent was obtained from all patients.

**Table 1:** Baseline data.*

Variables	Mean (SD)	Median
Age, years	64 (2.8)	64

Dry weight, kg	77.6 (18.3)	73.3

Hemoglobin, g/l	121 (4.7)	120

Albumin, g/l	37 (4.2)	38

Urea, mmol/l	21 (5.0)	22

Creatinine, μmol/l	683 (252)	625

total Cholesterol, mmol/l	4.0 (1.0)	4.1

LDL-cholesterol, mmol/l	1.9 (0.7)	1.9

HAL-cholesterol, mmol/l	1.2 (0.4)	1.1

Triglycerides, mmol/l	2.0 (1.1)	1.6

**Figure 1:**
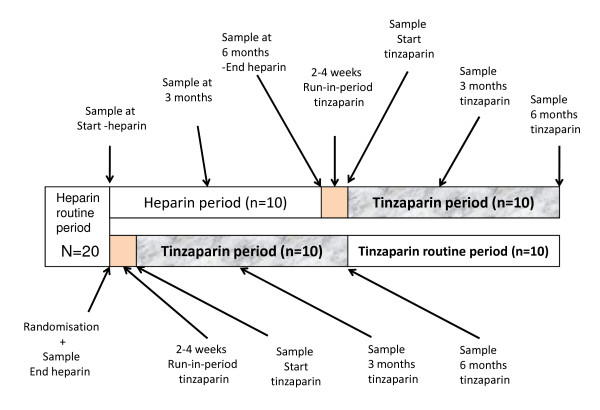
**Flow chart of the study**.

* The blood and serum values (± 1 standard deviation, SD) were obtained before dialysis at the last day of routine UF-heparin treatment and before start of the tinzaparin period (figure 1).

**Table 2:** Data for Kt/V, urea reduction rate, ultrafiltration volume and UF-heparin dose during the last performance of UF-heparin HD before start of the tinzaparin period.

Variables	Mean (SD)	Median
Kt/V	1.46 (0.32)	1.47

Urea reduction by dialysis, %	72.8 (5.5)	72.5

Ultrafiltration volume, liters	1.50 (1.10)	1.55

Ultrafiltration, % of body weight	2.1 (1.9)	2.0

UF-heparin total dose, Units	7573 (1533)	7500

The design of the study is illustrated in figure 1. After randomization six men and four women were switched to tinzaparin (Leo Pharma, Ballerup, Denmark). After a run in period they were followed for 6 months. Ten others (six men and four women) continued on UF-heparin for 6 months and then switched to tinzaparin and were followed for 6 months.

UF-heparin was given as a bolus of approximately 50 units/kg body weight at the start of HD followed by a continuous infusion of 800-1200 units/hour. The doses were adjusted with the aim to keep the activated partial thromboplastin time within 40-90 seconds until the last 45 min of dialysis. Then, the heparin infusion was stopped for those who had peripheral access, to allow sufficient coagulation upon stop of dialysis. For patients with a central dialysis catheter the infusion of heparin continued until the end of HD.

Tinzaparin (Leo Pharma, Ballerup, Denmark) was given as a bolus of 4500 anti-Xa units. After switching from heparin a run in period was allowed to adjust the tinzaparin dose, which usually took 2-4 weeks. If clotting was noticed (by vision) in the system or if there was a bleeding tendency, the dose was changed in steps of 500 units, according to the manufacturer's recommendations.

The dialysate contained conventional electrolytes (Meda, Solna, Sweden) and a final concentration of 5 mmol/l glucose. The dialyzers were from Fresenius Medical Care (Hechingen, Germany; F8HPS and FX80, one and four patients, respectively) and Gambro (Lund, Sweden; PF140 H and PF210 H, nine and six patients, respectively). Each patient used the same type of dialyzer for the whole series (no reuse).

Dry weight of the patient was followed throughout the study. This is the weight that a patient with normal urine production would have despite fluid intake. In patients with loss of urine output, water is retained between dialyses and weight increases (inter dialysis weight gain).

The body weight, the serum albumin and urea concentrations were stable over the observation period, indicating that the patients were not malnourished.

### Blood sampling and analytical procedures

The patients were asked to fast overnight if dialysis started in the morning. However, several refused fasting. Those who refused to fast (i.e. diabetic patients) were told to keep a low fat diet with a cup of tea and only carbohydrates for breakfast and to keep the same regime of diet for all dialyses included in the study. This was accepted by them.

After approximately 60 minutes of dialysis they received a light meal (sandwiches).

Blood samples before dialysis were obtained directly from the AV fistula or from the arterial line of the central dialysis catheter. Blood samples at the end of dialysis were drawn according to the Swedish guidelines for blood sampling after dialyses. This means that after the end of HD the dialysate flow is stopped and the blood flow is reduced to 100 ml/min for 15 seconds before the blood pump is stopped and disconnected from the patient. Most of the blood that resides in the tubes is returned to the patient. Blood samples were taken from the arterial needle at the site of the AV fistula or AV graft or drawn from the arterial part of the central dialysis catheter. When samples were taken during dialysis, they were drawn on the arterial side of the tubing set, before the dialyzer. This was the same in all series.

Urea was analyzed in blood samples drawn after the end of HD, to calculate urea reduction rate and Kt/V (according to the second generation formula by Daugirdas, considering the effect of the extent of ultrafiltration [10]). These parameters were used as measures for the weekly dose of dialysis to decide if the dialysis was efficient enough. An insufficient value would cause a prescription of more extensive dialysis. During this study such changes were not necessary. Urea reduction rate and Kt/V were also used as indirect markers of functional surface area of the dialyzer. If the surface was changed by clotting, the efficacy of dialysis would be reduced since the other variables were kept constant (blood flow, dialysate flow, dialyzer, dialysis time). In addition, the Transonic Flow-QC Hemodialysis Monitor (HD01 Plus, Transonic systems inc., NY, USA) was used to measure the blood volume of the dialyzer before and after dialysis, to estimate the extent of clotting that could appear during hemodialysis. A specific software for such calculations was provided by the manufacturer.

Blood samples were drawn during the dialyses (40, 180 and 210 min) for analysis of LPL and blood lipids. At the end of HD a sample was drawn for assay of Factor Xa.

LPL activity in the blood samples was measured as previously described after hepatic lipase had been removed by immunoadsorption [6].

Retrospectively we compared the serum concentrations of TG, total cholesterol, LDL, HDL, albumin and CRP during one year prior to the study and 1 1/2-2 years after the study. In three patients the post-study period was shortened due to transplantation. Four other patients died within one year after the end of the study period.

### Statistical analyses

Results are expressed as mean ± s.e.m. (standard error of mean). Statistical analysis was performed with SPSS software, version 11 (SPSS inc. Chicago, Illinois, USA). Paired non parametric (Wilcoxon) analyses were used to compare values measured for the same individual (n = 20 pairs). Non-paired statistics (Mann Whitney) were used to compare groups with each other. For analyses of correlation the Pearson test was used. A two tailed p-value of < 0.05 was considered as significant.

## Results

### UF-heparin/Tinzaparin doses

The median UF-heparin bolus was 2500 units (range 2000-4500) corresponding to 37 units/kg body weight. This was followed by a continuous infusion of a median of 1000 units/hour (range 200-1600 units/h), giving a total dose of 7500 units/dialysis (median, range 5200-10500). The change from UF-heparin to tinzaparin needed 0-4 weeks (based on visual evaluation of clotting or bleeding). Nineteen of the patients needed only one bolus of tinzaparin while one patient needed a second bolus (2000 units) after 180 min of dialysis (total dose 14000 units). Values at 210 min for this patient were excluded.

The median tinzaparin dose was 6000 units (range 3500-14000). This is in line with the dose of 75 anti-Xa units/kg body weight used in a detailed study of the pharmacokinetics of tinzaparin in patients undergoing HD [11-13].

The mean anti-Xa activity of the patients given tinzaparin was 0.26 U/L (± 0.05, range 0.07-0.78). The dialyzers remained subjectively patent by visual evaluation, during all dialyses. No patient had side effects due to anticoagulation, besides limited and expected local bleeding at the access after the end of the dialysis.

### Evaluation of dialysis efficacy

To estimate if clotting reduced the dialyzer volume, Transonic measurements were made at the start and at the end of dialysis. This showed that dialyzer volumes were not significantly reduced (median 1%, range -16 to + 10%). There were no differences in this regard between the two anticoagulants or between the dialyzers used.

There were also no differences in dry weight, serum creatinine, urea, albumin, change in filter area (measured by Transonic) or Kt/V comparing the two parallel groups (figure 1) that received UF-heparin or tinzaparin during the first 6 month period. Neither was there a difference when comparing, by paired statistics, each individual patient when on UF-heparin (last dose before switch) or tinzaparin (first dose when considered in steady state, after 2-4 w). The start values for Kt/V were 1.43 and 1.46 for the UF-heparin and tinzaparin measurements, respectively.

When comparing data during the 6 months tinzaparin follow-up period, urea reduction rate and Kt/V were lowered (p < 0.02) by 4% and 7% at 6 months (but not at 3 months), indicating a slight reduction in dialysis efficacy. There was no such reduction of these variables in the group that were studied for 6 month with UF-heparin, before switch to tinzaparin (figure 1).

### Lipoprotein lipase activity

**Figure 2:**
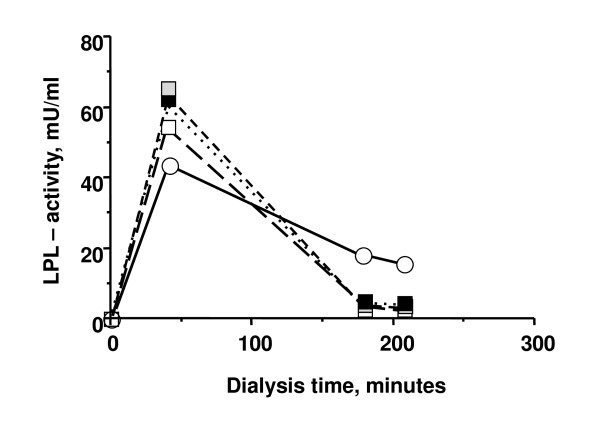
**Median LPL activities in plasma during dialysis with UF-heparin or tinzaparin**. UF-heparin - open circles and filled lines (at the points in time denoted "End heparin" in Figure 1); tinzaparin at start (i.e. after the run-in period) - open squares and hatched lines; tinzaparin 3 months - grey squares and hatched lines; tinzaparin 6 months - filled squares, dotted lines.

**Figure 3:**
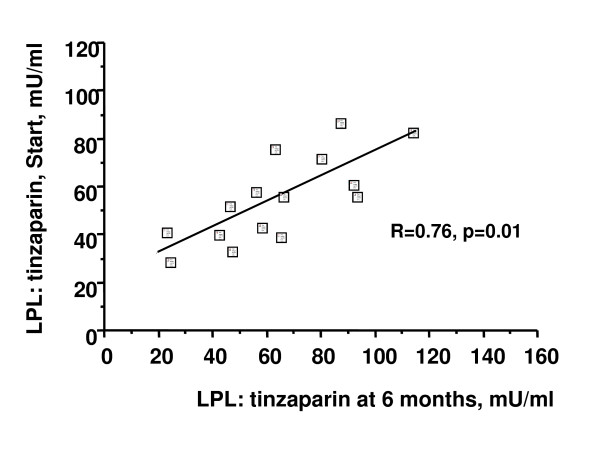
**Correlation of LPL- activity in plasma 40 minutes after injection of tinzaparin at the points in time designated in figure 1 as Start tinzaparin and 6 months tinzaparin, respectively**.

**Figure 4:**
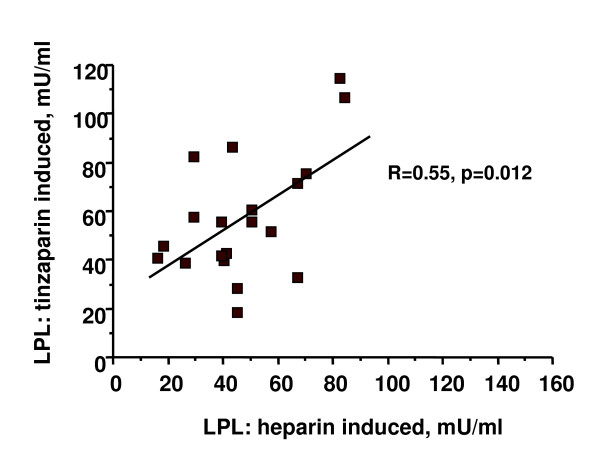
**Correlation of LPL- activity in plasma 40 minutes after injection of a bolus of tinzaparin or after start of a primed infusion of UF-heparin**. The values are from the points in time designated in figure 1 as Start heparin and Start tinzaparin, respectively

LPL activity in plasma was high at 40 min after injection of UF-heparin or tinzaparin and then decreased to much lower values at 180 min and 210 min (figure 2). No significant change occurred in the LPL activities over the 6 month study period whether they were tested as values at single times or as area under the curve. There was a strong correlation (r = 0.76, p < 0.01) between the peak LPL activity (40 min value) measured at the start of the tinzaparin period and after 6 months on tinzaparin (figure 3). Likewise there was a strong correlation between the peak LPL activities after tinzaparin and after heparin (r = 0.55, p < 0.02, figure 4).

The LPL activity at 40 min was somewhat higher after tinzaparin than after UF-heparin (figure 2 p < 0.05) whereas LPL activities after 180 and 210 min were lower using tinzaparin (figure 2 p < 0.001 at both times).

**Figure 5:**
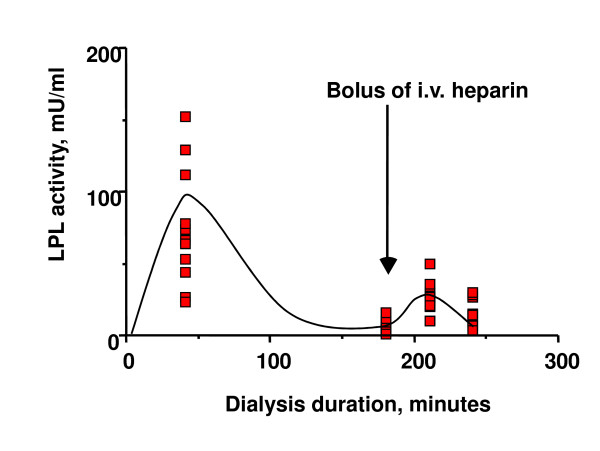
**Test of the amount of LPL available in the system at 40 minutes and during the end of a HD with tinzaparin**. A bolus of UF-heparin (50 units/kg body weight, intravenously) was given after 180 min of HD with tinzaparin to evaluate the extent of residual LPL on the endothelial sites. Eleven of the patients accepted to participate in this second step of investigation. Blood samples were taken at 180, 210 and 240 min (i.e. 0, 30 and 60 min after the bolus of UF-heparin).

To estimate the amount of LPL remaining in the tissues after HD with tinzaparin, a bolus of UF-heparin was given at 180 min to 11 patients (figure 5). Thirty min later, LPL activity had increased by 18 ± 8.0 U/l (p < 0.01). This increase was similar to that noted in a previous study with dalteparin as anticoagulant (19.5 ± 6.5 U/l) [5,14].

### Lipids

No significant changes occurred, neither during the study period using UF-heparin (figure 1), nor when all patients were switched to tinzaparin, in predialysis values for plasma TG, total cholesterol, LDL- or HDL- cholesterol, albumin or CRP. To further explore this we collected all data from the records for a period from one year before to 1.5 - 2 years after the study. Since there was a wide range of values among individual patients we calculated the ratio of the value while on tinzaparin divided by the value while on heparin. This returned the following ratios: TG 0.98 ± 0.09 (mean ± SEM); total cholesterol 0.97 ± 0.05; LDL cholesterol 0.94 ± 0.08; and HDL-cholesterol 1.00 ± 0.04. None of these ratios revealed any statistically significant change. The ratio for CRP was 1.45 ± 0.26 (p = 0.43).

**Figure 6:**
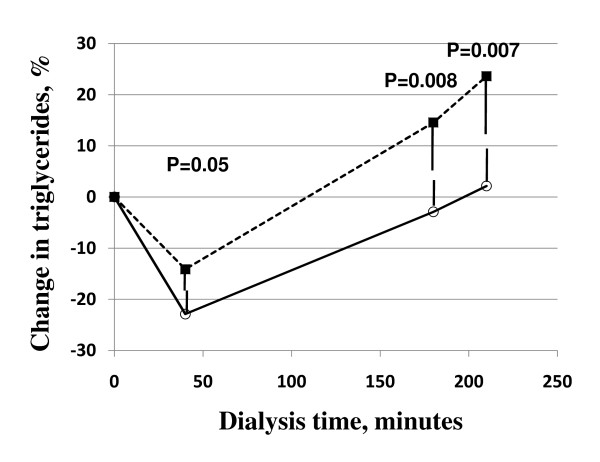
**Plasma triglyceride concentration during HD with UF-heparin (open circles and filled line) or tinzaparin (filled square and dotted line)**.

During the HD and after injection of UF-heparin or tinzaparin, plasma TG decreased at 40 min (p < 0.001), and then rose again to reach values at the end of the dialysis (180 and 210 min) similar to those before UF-heparin/tinzaparin (figure 6). The TG values did not decrease as much at 40 min and were then higher after tinzaparin than after UF-heparin at 180 and 210 min (p = 0.05, p < 0.01 and p < 0.01, respectively).

## Discussion

The main conclusion from this study is that long term repeated administrations either UF-heparin or tinzaparin during chronic hemodialysis does not cause exhaustion of the LPL production.

Regarding clinical practice there is a big advantage in the reduced time and material needed to prepare and administer tinzaparin instead of UF-heparin. However, we found a slight reduction in dialysis efficacy with tinzaparin at 6 months but not with UF-heparin, when comparing the values for the urea reduction rate and the Kt/V from start to the value obtained after 6 months with respective anticoagulant. We found no significant difference using the Transonic method (as a measure for patent capillaries of the dialyzer). The variation in data was larger with the Transonic method, and therefore the precision was lower than for urea reduction rate and Kt/V. Taken together these data indicate that the pore size may have decreased somewhat when using tinzaparin, perhaps by formation of a fibrin layer on the dialyzer surface. Our overall conclusion is, however, that there were no marked differences between the two anticoagulation regimes in their effect on the function of the dialyzers. The patients did not show any obvious changes in bleeding or clotting tendency, comparing the two heparin preparations. Our observations on the efficacy and safety of tinzaparin as anticoagulant in HD are in line with earlier studies [1,7-9,11-13,15]. Notable is, that in one of these studies the extent of clotting was increased in the venous bubble traps with tinzaparin [13], indicating an increased tendency of clotting.

In some studies the plasma LPL activity has been found to be lower after injection of LMW-heparin than after UF-heparin and this has led to the conclusion that LMW-heparin has a lesser impact on the LPL system. This is not correct. Studies in experimental animals have shown that LMW-heparins are at least as effective as UF-heparin to release the lipase into blood, but less effective in retarding hepatic uptake of the lipase [4]. When rat hearts were perfused by a single pass of medium, heparin decasaccharides (a molecular size that corresponds to the lower end of LMW-heparin preparations) released LPL into the medium more efficiently than UF-heparin [16]. In contrast, the decasaccharides had a poor ability to retard hepatic clearance of the lipase. Interpretation of time curves of lipase activity in blood after heparin injection is complex. In addition to the ability of the particular heparin preparation to release the lipase and to retain it in the circulating blood one needs to consider the number of heparin molecules injected (rather than coagulation-based units), their size and sulfation, as well as the rate at which different heparin molecules are cleared (longer heparin chains are cleared more rapidly). In the present study plasma LPL activity tended to be somewhat higher 40 min after tinzaparin than after UF-heparin. This presumably reflects that a larger number of heparin molecules were injected with the LMW preparation and that these molecules efficiently released the lipase. It is of interest to note that there was a strong correlation between the LPL activites 40 min after tinzaparin and after heparin in individual subjects. After 180 min the plasma LPL activity was lower with tinzaparin, presumably reflecting a lesser ability of the shorter heparin chains to retain the enzyme in the circulating blood. In an earlier study by Näsström et al. the peak LPL activity after dalteparin was only about one-third of that with UF-heparin. A difference between the studies is the use of tinzaparin instead of dalteparin that was used in the study by Näsström et al. [6]. Tinzaparin is known to have the largest mean molecular weight of all LMW-heparins [2] and is therefore structurally closer to UF-heparin. This probably explains the different patterns of plasma LPL activity during HD with dalteparin or tinzaparin.

The effect of heparin injection on lipoprotein metabolism is biphasic [3]. Initially there is accelerated lipolysis. The interpretation is that LPL has more ready access to substrate lipoproteins when it is in the circulating blood. In the basal state the lipase is at binding sites at the vascular endothelium [17], whereas most of the lipoproteins are in the circulating blood. Only a fraction of the lipoprotein particles are "marginated" at the endothelium, in contact with LPL [18]. This early phase of high LPL activity in the circulating blood is reflected by decreased levels of TG [5,19,20]. In agreement with Akiba et al [21] and Katopodis et al [19] we found that this early fall of plasma TG was less marked with the LMW-heparin than with the UF-heparin. There was a similar trend in the earlier study by Näsström et al [5] but in that case the difference did not reach statistical significance. The mechanism behind this is not immediately apparent. The higher levels of LPL activity in blood at 40 min likely results in more rapid lipolysis of lipoprotein triglycerides in the circulating blood. However, heparin also accelerates removal of partially lipolyzed lipoprotein particles [3,22] and the heparin preparations may differ in this regard, with UF-heparin being more efficient than LMW-heparin.

With time after the administration of heparin, accelerated transport of LPL from its natural sites of action to the liver (where it is degraded [23]) leads to a temporary depletion of the lipase [4,14]. Evidence for this in the present study was the low amounts of LPL that could be recruited into plasma by a second injection of a heparin bolus late in the HD. This loss of LPL from the system is presumably the main reason that plasma TG tend to increase towards the end of the HD session and during the hours thereafter [5]. This increase was more marked with tinzaparin than with UF-heparin similar to earlier studies with dalteparin [5] and with enoxaparin [20]. This indicates that during the later part of the HD and some hours thereafter, the system is more depleted of LPL when using a LMW-heparin than when using UF-heparin. The reduction of LPL at the endothelial sites (in the early phase of administration) and the loss of active LPL (low levels during later part of HD) seems to deplete ability of the patient to degrade triglycerides into the energy resource of free fatty acids, during this later phase of the HD. Apparently the anticoagulation regimes do not result in a persistent depletion of LPL, since the initial peak value after tinzaparin injection remained unchanged throughout the 6 month study period. Hence, the system was able to fully recover between dialyzes. Likewise, the temporarily decreased ability to catabolize plasma TG, indicated by the rising plasma TG levels, did not seem to carry over to a persistent derangement of lipoprotein metabolism, since the plasma lipid levels did not change during the 6 months study period. Again, the system was apparently able to fully recover between dialyses.

Elevated plasma lipid concentrations, particularly TG, is a common concern in patients on chronic HD, since cardiovascular diseases are the main causes of death in patients on chronic HD. Therefore, the effects of different heparin preparations on plasma lipid levels (measured between dialyses) are of interest. This has been studied in a number of laboratories with somewhat varying results. It has been suggested that UF-heparin may lead to aggravated hyperlipidemia during long term treatment in patients on chronic HD [24,25]. In most studies LMW-heparin during HD is associated with decreases of TG and of total and LDL-cholesterol [26-29]. These decreases appear to be more pronounced in patients with hyperlipidemia [21,30-33], which is common in patients with renal disease. More detailed studies report a shift of LDL particles towards more buoyant species away from the more atherogenic small dense LDL subfractions [33]. These changes of the lipoprotein profile with LMW-heparins may be regarded as beneficial with respect to development of atherosclerosis. There are, however, also large, well-controlled studies that have found no consistent effect of LMW-heparin on plasma lipids [34] and there are even studies that report increased plasma lipid levels while on HD with LMW-heparin [35]. In the present study we did not observe any significant changes of the plasma lipid levels studied, even when comparing values measured during one year prior to the study and values measured during 1 1/2-2 years after the study.

## Conclusion

Our data suggest that repeated HD with UF-heparin or tinzaparin does not exhaust the LPL-system.

## List of Abbreviations

HD: hemodialysis; HDL: high density lipoprotein; LDL: low density lipoprotein; LMW-HEPARIN: low molecular weight heparin; LPL: lipoprotein lipase; UF-HEPARIN: unfractionated heparin; TG: triglycerides; AV: arterio-venous; KT/V: calculation of weekly dialysis dose performed; LDL: low density lipoprotein cholesterol; HDL: high density lipoprotein cholesterol; CRP: C-reactive protein.

## Competing interests

The authors declare that they have no competing interests.

## Authors' contributions

DM participated in the sequence alignment and drafted the manuscript. MG participated in the sequence alignment and sampling procedure. LL participated in the design of the study. GO participated in the design of the study and of the sequence alignment. TO participated in the design of the study and performed some statistical analysis. BS conceived the study, and participated in its design and coordination and performed statistical analysis. All authors read and approved the final manuscript.

## Pre-publication history

The pre-publication history for this paper can be accessed here:

http://www.biomedcentral.com/1471-2369/11/33/prepub
